# Isolation and Synthesis of a Bioactive Benzenoid Derivative from the Fruiting Bodies of *Antrodia camphorata*

**DOI:** 10.3390/molecules18077600

**Published:** 2013-06-28

**Authors:** Pi-Yu Chen, Jen-Der Wu, Kai-Yih Tang, Chieh-Chou Yu, Yueh-Hsiung Kuo, Wen-Bin Zhong, Ching-Kuo Lee

**Affiliations:** 1 School of Pharmacy, Taipei Medical University, Taipei 110, Taiwan; E-Mails: e023089103@tmu.edu.tw (P.-Y.C.); jd0331@tmu.edu.tw (J.-D.W.); 2 Yong Chung Prosperous Biotech Co. Ltd., New Taipei 231, Taiwan; E-Mail: xz029465@gmail.com; 3 Twherb Biomedical Co. Ltd., Jhubei 302, Taiwan; E-Mail: 123@twherb.com.tw; 4 Tsuzuki Institute for Traditional Medicine, College of Pharmacy, China Medical University, 404 Taichung, Taiwan; E-Mail: yhkuo@ntu.edu.tw; 5 School of Medicine, Taipei Medical University, Taipei 110, Taiwan

**Keywords:** *Antrodia camphorata*, enynyl-benzenoid, antrocamphin O, synthesis, cytotoxic activity

## Abstract

A new enynyl-benzenoid, antrocamphin O (1,4,7-dimethoxy-5-methyl-6-(3′-methylbut-3-en-1-ynyl)benzo[d][1,3]dioxide), and the known benzenoids antrocamphin A and 7-dimethoxy-5-methyl-1,3-benzodioxole, were isolated from the fruiting bodies of *Antrodia camphorata* (*Taiwanofungus camphoratus*). The structure of antrocamphin O was unambiguously assigned by the analysis of spectral data (including 1D and 2D NMR, high-resolution MS, IR, and UV) and total synthesis. Compound **1** was prepared through the Sonogashira reaction of 5-iodo-4,7-dimethoxy-6-methylbenzene and 2-methylbut-1-en-3-yne as the key step. The benzenoids were tested for cytotoxicity against the HT29, HTC15, DLD-1, and COLO 205 colon cancer cell lines, andactivities are reported herein.

## 1. Introduction

*Antrodia camphorata* (*Taiwanofungus camphoratus*), also known as “Niu-chang-chih,” belongs to the familyPolyporaceae in the Basidiomycetes. Designated an endangered species in Taiwan, the fungus is a rare and unique mushroom used in folk medicine and as a health food. To date, over 200 constituent compounds, consisting of small molecules (terpenoids, benzenoids, lignans, benzoquinone derivatives, and succinic and maleic derivatives) and macromolecules (polysaccharides, nucleic acids, and proteins), have been reported [1,2,3]. Preparations from the fruiting bodies have been used for the prevention or treatment of numerous maladies, including liver diseases, food and drug intoxication, diarrhea, abdominal pain, hypertension, itchy skin, and tumorigenic diseases [4]. Previous research has shown the anti-cancer and anti-inflammatory effects of the triterpenoids derived from *A. camphorate* [3,5]. The major chemical constituents, the enynyl-benzenoids, are the key components responsible for anti-inflammatory activity [6,7]. Because the growth rate of *A.camphorata* in both the wild and under cultivation is very slow, the fruiting bodies are rare and expensive. Therefore, synthesis of the active compounds is very important. In this paper, we isolated and elucidated the structure of a new active compound, the enynyl-benzenoid antrocamphin O (**1**), and the known benzenoids, antrocamphin A (**2**) [8] and 4,7-dimethoxy-5-methyl-1,3-benzodioxole (**3**) [9], from the fruiting bodies of *A*. *camphorata*. We also describe the total synthesis of **1**. The benzenoids were tested for cytotoxicity against the HT29, HTC15, DLD-1, and COLO 205 colon cancer cell lines, and their activities are reported herein.

## 2. Results and Discussion

### 2.1. Isolation and Identification

**Figure 1:**
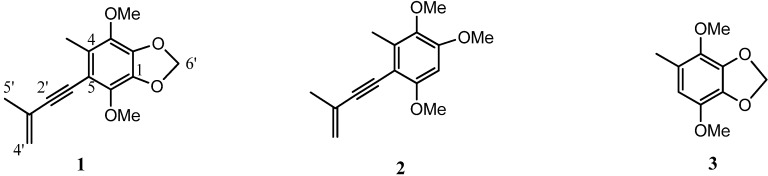
Structures of **1–3** from the fruiting bodies of *A*. *camphorate*.

The EtOH extract of the fruiting bodies of *A. camphorata* (48 g, dried) was subjected to liquid-liquid partitioning with EtOAc, BuOH, and H_2_O. The bioactive EtOAc-soluble fraction was chromatographed over silica gel, followed by HPLC separation using refractive index detection. This procedure afforded three compounds **1−3**. The structural elucidation of compound **1** was carried out using spectroscopic analysis and 2D NMR techniques, including ^1^H-^1^H COSY, heteronuclear multiple quantum correlation (HMQC), and heteronuclear multiple bond correlation (HMBC) experiments. Compound **1** was obtained as a colorless amorphous powder. The molecular formula was established as C_15_H_16_O_4_ by high-resolution electron spray ionization MS (HRESIMS, *m*/*z* 261.1123 [M+H]^+^, calcd for C_15_H_17_O_4_, 261.1127). The UV spectrum of 1 displayed absorption maxima at 258 and 288 nm, and the IR spectrum exhibited characteristic absorption peaks for alkynes (2183 cm^−1^) and alkenes (1605 cm^−1^). The ^1^H-NMR (CDCl_3_) data for **1** (Table 1) showed terminal methylene protons at δ 5.35 (1H, br s, H-4'a) and δ 5.24 (1H, br s, H-4′b), two methoxy protons at δ 3.85 (3H, H-3) and 3.96 (3H, H-6), and two methyl singlets at δ 2.25 (3H, s, 4-Me) and δ 1.98 (3H, br s, H-5'). The ^13^C-NMR data for **1** (Table 1) indicated 15 carbon resonance signals, in agreement with the HRESIMS data, including olefinic peaks at δ 121.0 (C-4′) and δ 127.2 (C-3′), two methoxy groups at δ 60.0 (3-OMe) and δ 60.3 (6-OMe), two methyl groups at δ 13.8 (4-Me) and δ 23.6 (C-5′), an sp^3^ methylene at δ 101.4, and other eight quaternary carbons (83.5, 97.5, 109.9, 127.8, 136.3, 137.2, 139.5, and 139.8). The structure of **1** was assigned by 2D NMR data (COSY, HSQC, and HMBC). A ^1^H-^1^H COSY correlation for H-5′/H-4′ and HMBC cross-peaks of H-5′ to C-2′, C-3′, and C-4′ suggested the presence of an enynyl moiety. From 3-OCH_3_ (δ 3.85) to C-3, 4-CH_3_ (δ 2.25) to C-3/C-4/C-5, and 6-OCH_3_ (δ 3.96) to C-6 by HMBC experiment, the chemical structure of **1** was identified to be as shown in Figure 1. This is the first report from natural sources of this compound, which was given the trivial name antrocamphin O. 

**Table 1:** NMR Spectroscopic Data for **1** in CDCl_3_ (500 MHz).

**Position**	**Antrocamphin O (1)**		
	**δ_C_**	**δ_H_ (*J* in Hz)**	**HMBC**
1	136.3		
2	139.5		
3	137.2		
4	127.8		
5	109.9		
6	139.8		
1′	83.5		
2′	97.5		
3′	127.2		
4′	121.0	5.24 (br s), 5.35 (br s)	2′, 3′, 5′
5′	23.6	1.98 (br s)	2′,3′, 4′
6′	101.4	5.91 (s)	1, 2
3-OMe	60.0	3.85 (s)	3
6-OMe	60.3	3.96 (s)	6
4-Me	13.8	2.25 (s)	3, 4, 5

### 2.2. Synthesis and Structural Characterization of Antrocamphin O (1)

**Scheme 1:**
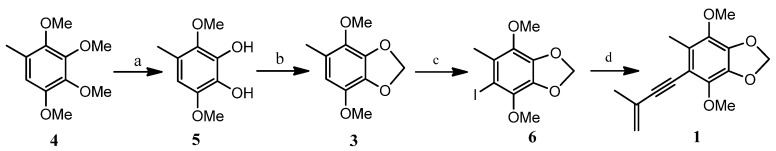
The synthetic route to compound **1**.

To establish the proposed structure of **1** unambiguously, a sample of reference material was needed, and this prompted us to prepare the authentic compound. The reaction sequence outlined in Scheme 1 was followed. Treatment of the readily available 2,3,4,5-tetramethoxytoluene (**4**) with AlCl_3_ followed by demethylation afforded **5** and its isomers [10]. Purification of the isomeric mixture by column chromatography gave **5** in an overall yield of 34%. The NMR data analysis (^1^H, ^13^C-NMR, and nOe) revealed the exact structure of **5**. After protection with bromochloromethane in the presence Cs_2_CO_3_ at 110 °C [11], the 1,3-benzodioxole **3** was obtained in 70% yield. Iodination of **3** with *N*-iodosuccinimide [12] produced 5-iodo-4,7-dimethoxy-6-methylbenzo[d][1,3]dioxole **6**. The Sonogashira reaction [13] was utilized to couple iodide **6** with 2-methyl-1-buten-3-yne, using the catalysts Pd(PPh_3_)_4_ and CuI. This key reaction led to the desired compound, antrocamphin O (**1**).

*Reagents and conditions*: a, AlCl_3_, 40 °C, 34%; b, CH_2_ClBr, Cs_2_CO_3_, 110 °C, 70%; c, *N*-iodosuccinimide, 37 °C, 88%; d, Pd(Ph_3_)_4_, CuI, Et_3_N/THF, 90 °C, 16%.

### 2.3. Cytotoxicity Assay

**Figure 2:**
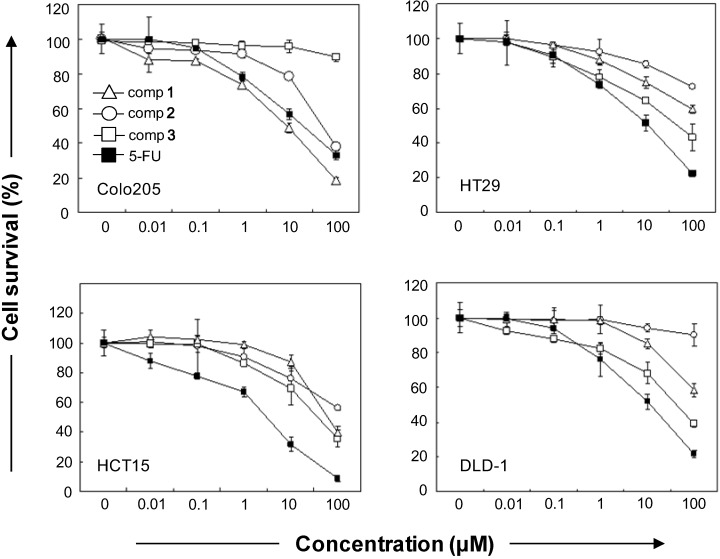
Effects of AC1-derived compounds on cell survival inhibition. Colon cancer cells were either treated with compound **1–3**, or 5-FU for 48 h. MTT test was used to exam the relative cell number. **1–3**, and 5-FU concentration-dependently inhibited the cell viability in 4 clones of colon cancer cells. Values represent the means ± S.E.M. (n = 3). Significance was accepted at *p* < 0.05.

The cytotoxicity evaluation of compounds **1–3** against four human cancerous cell lines (HT29, HTC15, DLD-1, and COLO 205) showed that **1–3** were moderately or less active than 5-fluorouracil (5-FU), except against the COLO 205 cell line (Figure 2). Antrocamphin O (**1**) was a potent inhibitor of COLO 205 colon cancer, with higher activity than the reference compound 5-FU (Table 2). 

**Table 2:** The IC_50_ cytotoxicity of compounds **1–3** and 5-FU toward different colon cancer cells.

**Compound**	**IC_50_ (µM)**			
	**HT29**	**HTC15**	**DLD-1**	**Colo205**
**1**	>100	70	>100	9.3
**2**	>100	>100	>100	43.4
**3**	14	39	40	>100
**5-FU**	11.2	3.1	9.5	17

## 3. Experimental

### 3.1. General Methods

IR spectra were recorded on a JASCO 4100 FT-IR spectrometer. NMR data were recorded on a Bruker DRX-500 SB 500 MHz instrument (Bruker, Rheinstetten, Germany) with CDCl_3_ as solvent and TMS as internal standard. ESIMS and HRESIMS data were acquired using an ABI API 4000Q Trap and ABI XL Q-TOF mass spectrometer (Applied Biosystems, Foster City, CA, USA), respectively. Semi-preparative HPLC was performed on a Hitachi L-7110 HPLC with a refractive index detector (Thermo Separation Products, Sunnyvale, CA, USA). A Phenomenex Luna silica column (5 μm, 10 × 250 mm, 3 mL·min^−1^ flow rate) was used for normal-phase separations. Silica gel (Merck, Geduran^®^ Si 60 0.063–0.2 mm) was used for column chromatography. All solvents were either ACS or HPLC grade and were obtained from JT Baker (Phillipsburg, NJ, USA). 5-FU was purchased from Sigma-Aldrich (St. Louis, MO, USA).

### 3.2. Plant Material

The fruiting bodies of *Antrodia camphorata* were collected in Taoyuan, Taiwan, in the summer of 2009, and kindly provided to our laboratory by the Cosmos Biotech. Company (Longtan, Taiwan). The sample was authenticated by Tien-Wang Chiu (general manager of Cosmos Biotech. Company), and a voucher specimen (AC2009-1) was deposited at the School of Pharmacy, Taipei Medical University, Taipei, Taiwan.

### 3.3. Extraction and Isolation

*A. camphorata* (48 g) were cut into small pieces and extracted with 4 L ethanol at room temperature for seven days. After removal of the solvent under reduced pressure, the ethanolic extract residue was separated by silica gel column chromatography with *n-*hexane, ethyl acetate, and methanol. Fractions of 100 mL each were collected and monitored by TLC using *n*-hexane/ethyl acetate (4:1 and 3:1, v/v) or *n*-hexane/acetone (3:1, v/v) and observed at 254 nm. Fractions with similar components were pooled into 25 portions. Silica gel column chromatography of portions 1–4 (1.5 g) comprised stepwise elution with *n-*hexane/ethyl acetate (99:1, 98:2, and 95:5, v/v), and finally, with pure ethyl acetate. Subfractions 12–18 were separated by normal-phase HPLC on a semipreparative normal phase column (Phenomenex Luna silica, 5 μm, 10 × 250 mm) at a flow rate of 3 mL·min^−1^, with a mixture of *n*-hexane/ethyl acetate (49:1, v/v), to afford compounds 3 (320 mg, t_R_ = 23.9 min) and **1** (12 mg, t_R_ = 26.5 min). Compound **2** (39 mg, t_R_ = 11.9 min) was obtained from subfractions 26–28 by HPLC with an isocratic mixture of *n*-hexane/acetone (30:1, v/v), and then, with *n*-hexane/ethyl acetate/acetone (60:1:1, v/v/v).

*Antrocamphin O* (**1**): colorless amorphous powder; UV (MeOH) λ_max_(log ε) 213 (4.52), 258 (3.61), 288 (3.96) nm; IR (neat) ν_max_ 2942, 2183, 1605, 1469, 1449, 1276, 1051 cm^−1^; ^1^H- and ^13^C-NMR data, see Table 1; ESIMS (positive) *m/z* 261 [M+H]^+^; HRESIMS (positive) *m*/*z* 261.1123 [M+H]^+^ (calcd. for C_15_H_16_O_4_ 261.1127).

*Antrocamphin A* (**2**): ESIMS (positive) *m/z* 247 [M+H]^+^; ^1^H and ^13^C-NMR data, see reference [8]. 

*4,7-Dimethoxy-5-methyl-1,3-benzodioxole* (**3**): ESIMS (positive) *m/z* 197 [M+H]^+^; ^1^H and ^13^C-NMR data, see reference [9].

### 3.4. Synthesis

*Synthesis of 3,4-dihydroxy-2,5-dimethoxytoluene* (**5**). To a solution of 2,3,4,5-tetramethoxytoluene **4** (0.94 g, 4.43 mmol) in anhydrous dichloromethane (12 mL) was added AlCl_3_ (1.30 g, 9.77 mmol) under nitrogen. The mixture was heated to 40 °C for 16 h. After cooling to room temperature, the mixture was poured into iced water (60 mL) and extracted with dichloromethane. The organic layer was washed with saturated NaCl solution, and was then purified by column chromatography (eluent: ethyl acetate/hexane = 1:4 to 1:1) to afford pure 5 as a yellowish oil (0.28 g, yield: 34%). TLC R_f_: 0.28 (EtOAc:hexane 1:1); ^1^H-NMR (CDCl_3_) δ 2.22 (s, 3H), 3.78 (s, 3H), 3.82 (s, 3H), 5.48 (br s, 1H), 5.66 (br s, 1H), 6.24 (s, 1H); ^13^C-NMR (CDCl_3_) δ 15.5, 56.2, 60.6, 104.0, 120.9, 131.5, 137.1, 140.3, 143.4.

*Synthesis of 4,7-dimethoxy-5-methyl-1,3-benzodioxole* (**3**). To a solution of 5 (240 mg, 1.35 mmol) dissolved in dimethyl sulfoxide (3 mL) were added bromochloromethane (227 mg, 1.76 mmol) and Cs_2_CO_3_ (442 mg, 1.35 mmol). The mixture was then heated to 110 °C under nitrogen for 16 h. The mixture was cooled and then diluted by the addition of water (15 mL). The mixture was extracted with isopropanol (15 mL), and the extract was washed with saturated NaCl solution. A dark brown raw product (250 mg) was obtained, which was purified by column chromatography (eluent: ethyl acetate/hexane = 1:5), affording **3** as a colorless liquid (85 mg, yield: 70%). TLC R_f_: 0.52 (EtOAc:hexane 1:2); ^1^H-NMR (CDCl_3_) δ 2.17 (s, 3H), 3.84 (s, 3H), 3.88 (s, 3H), 5.93 (s, 2H), 6.30 (s, 1H); ^13^C-NMR (CDCl_3_) δ 15.8, 56.8, 59.8, 101.3, 108.8, 123.5, 134.6, 136.5, 138.6, 138.8; ESIMS (positive) *m*/*z*: 197 [M+H]^+^.

*Synthesisof* *5-iodo-4,7-dimethoxy-6-methyl-1,3-benzodioxole* (**6**). To a solution of **3** (240 mg, 1.35 mmol) dissolved in bromochloromethane (2 mL) was added *N*-iodosuccinimide (217 mg, 0.97 mmol). The reaction was heated to 37 °C and allowed to proceed overnight. The mixture was cooled and then diluted with water (5 mL). The mixture was extracted with CH_2_Cl_2_ (6 mL), then washed with 10% Na_2_S_2_O_3_ solution (10 mL). The product, **6**, was obtained as a light-orange oil (248 mg, yield: 88%). ^1^H-NMR (CDCl_3_) δ 2.34 (s, 3H, -CH_3_), 3.87 (s, 3H, -OCH_3_), 3.92 (s, 3H, -OCH_3_), 5.96 (s, 2H, -OCH_2_O-); ^13^C-NMR (CDCl_3_) δ 21.4, 60.1, 60.2, 87.9, 101.6, 127.7, 136.2, 137.3, 138.3, 139.6.

*Synthesis of* *4,7-dimethoxy-5-methyl-6-(3-methylbut-3-en-1-ynyl)benzo[d]*[1,3]*dioxole* (1). *N,N*-Dimethylacetamide (DMA, 1.0 mL) was placed in a 10 mL flask and heated to 60 °C for 20 min while purging with argon gas. To the flask was added 6 (230 mg, 0.71 mmol), copper iodide (130 mg, 0.71 mmol), Pd(PPh_3_)_4_ (130 mg, 0.07 mmol), and triethylamine (0.15 mL), and the reaction was heated to 80 °C. A solution of 2-methylbut-1-en-3-yne (192 mg, 2.5 mmol) was dissolved in DMA (0.5 mL) and added to the reaction mixture. The mixture was heated at 90 °C for 16 h. The mixture was cooled, and then, it was filtered after adding 15 ml of EtOAc. The filtrate was washed with H_2_O (6 mL). A brown product was obtained after concentration *in vacuo*. The raw product was purified by column chromatography (eluent: ethyl acetate/hexane = 1:6) to afford **1** as a colorless liquid (30 mg, yield: 16%). ^1^H-NMR (CDCl_3_) δ 2.01 (s, 3H, -CH_3_), 2.27 (s, 3H, Ar-CH_3_), 3.87 (s, 3H, -OCH_3_), 3.98 (s, 3H, -OCH_3_), 5.26 (s, 1H), 5.37 (s, 1H), 5.93 (s, 2H, -OCH_2_O-); ^13^C NMR (CDCl_3_) δ 13.8, 23.5, 60.0, 60.3, 83.5, 97.5, 101.4, 109.9, 121.0, 127.2, 127.8, 136.3, 137.2, 139.5, 139.8.

### 3.5. *In Vitro* Cytotoxicity Assay

#### 3.5.1. Cell Culture

Human colon cancer cells (HCT15, HT29, DLD-1, and COLO 205) were cultured in Dulbecco’s modified Eagle medium supplemented with 10% fetal bovine serum and antibiotics (100 U/mL penicillin and 100 mg/mL streptomycin) at 37 °C in a CO_2_ incubator. Cells grew to 90% confluence, were harvested with trypsin/EDTA, and sub-cultured in a new tissue culture flask after removing trypsin and EDTA. The cancer cells used in this study had passage numbers of less than ten.

#### 3.5.2. Cytotoxicity Assay

Colon cancer cells (1 × 10^5^ cells) were seeded into 96-well plates and cultured overnight, allowing cell attachment. This was followed by treatment with compounds **1**, **2**, and **3** for 48 h at various concentrations (from 0.001 to 100 μM), as indicated, and 5-FU as a positive control. At the end of the experiment, the cytotoxicities of the compounds were examined by the MTT ((3-(4,5-dimethylthiazol-2-yl)-2,5-diphenyltetrazolium bromide) test. MTT solution (400 mg/mL) was added into the cultured wells and incubated for 1 h. The medium was then aspirated, and the dark blue crystal product was extracted with DMSO. Colorimetric changes were read on a microtiter plate reader with a 570 nm filter and a reference wavelength of 430 nm. 

#### 3.5.3. Statistical Analysis

All data were expressed as the mean value ± S.E.M. Comparisons were subjected to the Student’s two-tailed t test. Significance was accepted at *p* < 0.05.

## 4. Conclusions

In conclusion, we have achieved the first total synthesis of the rare new natural product, antrocamphin O (**1**,), using the Sonogashira reaction as the key step. The intermediate **3**, from the fruiting bodies of *A. camphorata*, was also successfully synthesized during the process (Scheme 1). Whereas enynyl-benzenoids are considered the key components responsible for the anti-inflammatory activities demonstrated by these fungi, this paper offers the first report of the inhibition of COLO 205 cancer cells by such compounds too [13].
